# Systematic profiling of diagnostic and prognostic value of autophagy-related genes for sarcoma patients

**DOI:** 10.1186/s12885-020-07596-5

**Published:** 2021-01-12

**Authors:** Yuanhe Wang, Jianyi Li, Cheng Shao, Xiaojie Tang, Yukun Du, Tongshuai Xu, Zheng Zhao, Huiqiang Hu, Yingyi Sheng, Chuan Hu, Yongming Xi

**Affiliations:** 1 Department of Orthopaedic Surgery, The Affiliated Hospital of Qingdao University, Qingdao, 266071 China; 2 Department of Spinal Surgery, Yantai Affiliated Hospital of Binzhou Medical University, Yantai, 264100 China

**Keywords:** Autophagy-related genes, Sarcoma, Immune cell, Immune checkpoint, Prognosis

## Abstract

**Background:**

Autophagy-related genes (ARGs) have been confirmed to have an important role in tumorigenesis and tumor microenvironment formation. Nevertheless, a systematic analysis of ARGs and their clinical significance in sarcoma patients is lacking.

**Methods:**

Gene expression files from The Cancer Genome Atlas (TCGA) database and Genotype-Tissue Expression (GTEx) were used to select differentially expressed genes (DEGs). Differentially expressed ARGs (DEARGs) were determined by matching the DEG and HADb gene sets, which were evaluated by functional enrichment analysis. Unsupervised clustering of the identified DEARGs was conducted, and associations with tumor microenvironment (TME), immune checkpoints, and immune cells were analyzed simultaneously. Two prognostic signatures, one for overall survival (OS) and one for disease-free survival (DFS), were established and validated in an independent set.

**Results:**

In total, 84 DEARGs and two clusters were identified. TME scores, five immune checkpoints, and several types of immune cells were found to be significantly different between two clusters. Two prognostic signatures incorporating DEARGs showed favorable discrimination and were successfully validated. Two nomograms combining signature and clinical variables were generated. The C-indexes were 0.818 and 0.747 for the OS and DFS nomograms, respectively.

**Conclusion:**

This comprehensive analyses of the ARG landscape in sarcoma showed novel ARGs related to carcinogenesis and the immune microenvironment. These findings have implications for prognosis and therapeutic responses, which reveal novel potential prognostic biomarkers, promote precision medicine, and provide potential novel targets for immunotherapy.

## Background

Sarcoma is a group of malignant tumors originating from stromal cells, which can be divided into soft tissue sarcoma (including muscle, fat, nerve, blood vessel and other connective tissue) and bone sarcoma [1]. According to the report of cancer statistics, the total incidence of sarcoma is close to 5 per 100,000 people per year, accounting for 15% of all malignant tumors in children and 1% of malignant tumors in adults [2]. With the development of diagnostic and therapeutic technology, the 5-year survival rate was higher than 70% in some sarcomas. However, as a whole, the improvement in the diagnosis and treatment for sarcoma is still slowed down [3, 4]. For advanced stage patients or patients with distant metastasis, the 5-year survival rate was lower than 20% [4]. Although some strengthening therapies or new drugs were used for treating sarcoma patients, the outcome were still unsatisfactory [5, 6]. Therefore, developing effective tools for early diagnosis and prognostic prediction plays a vital role in the tailor management of sarcoma patients. Nowadays, AJCC TNM staging system is widely used to evaluate the prognosis of tumors and guide treatment decisions. However, the accuracy of TNM staging is not satisfactory and the prognosis of patients with same TNM stage may be various.

Autophagy is a metabolic and circulatory process in which cells decompose damaged organelles and proteins and release decomposition products [7]. It has been reported that autophagy regulates many pathological and physiological processes, such as cell differentiation and death, immune stress, inflammatory reaction, and anti-aging [7–11]. Nevertheless, autophagy had dual effects in the process of tumorigenesis and tumor progression. In the early stages of tumorigenesis, autophagy can suppress tumor probably by reducing cell damage and chromosome instability [12]. However, for existing tumor, autophagy can promote tumor progression by releasing decomposition products and providing nutrition for tumor cells [13]. Recent study indicated that several autophagy-related genes (ARGs) affect the activation of autophagy and the mutation of ARGs play an important role in the pathogenesis of cancer [14–17]. Moreover, ARGs may have potential value for prognostic prediction for tumor patients, including sarcoma. For example, Zhao et.al reported that the positive expression of ATG5 associated with TSSC3 can inhibit the metastasis and invasion of osteosarcoma cells [18]. In addition, Muscolino et.al demonstrated that the inhibition of ARG expression was related to the immune escape of Kaposi’s sarcoma-associated herpesvirus [19]. Although the role of autophagy in the occurrence and development of sarcoma were initially reported in present studies, most studies have focused on specific ARGs and no research performed a comprehensive analysis of ARGs for sarcoma.

In this study, based on a series of available data sets, we performed a comprehensive analysis of ARGs to explore the diagnostic and prognostic values of ARGs for sarcoma patients. Meanwhile, two ARGs-based prognostic nomograms were established and externally validated and the association between ARGs and tumor microenvironment were studied.

## Methods

### Data acquisition and processing

The RNA-sequencing data of 911 normal human muscle and adipose tissues and 259 primary sarcoma were obtained from the UCSC Xena browser (https://xenabrowser.net/). For both normal tissue and sarcoma cohorts, RNA-sequencing data (FPKM values) were normalized into log2 (FPKM+ 1). Meanwhile, the corresponding clinical data were downloaded from the UCSC Xena browser and cBioPortal (http://www.cbioportal.org/). Meanwhile, for independent cohorts, including GSE2719, GSE21122, GSE30929, and TARGET-OS, were included as validation cohorts. The first two cohorts were used to validate the diagnostic value of ARGs for sarcoma, while the remaining cohorts were used to validate the prognostic value of ARGs for sarcoma. The expression file and clinical data of TARGET-OS cohort were downloaded from the UCSC Xena browser, whereas the data of GSE2719, GSE21122, and GSE30929 cohorts were obtained from the Gene Expression Omnibus database (https://www.ncbi.nlm.nih.gov/geo).

### Profiling of DEARGs for sarcoma

To determine sarcoma-related genes, differential analyses were performed between tumor samples and normal tissues with “limma” package. We used R software to perform Wilcoxon signed-rank test to identify the differential expression genes (DEGs). The cutoff value of DEARGs was identified by | log2 (FC) | > 1 and adjusted *p* value < 0.05. Furthermore, according to the ARGs list obtained from the HADb (http://autophagy.lu/clustering/index.html), DEARGs were confirmed. Heatmap and circos plot were used to visualize DEGs and DEARGs.

Furthermore, to understand the function and related pathway of DEARGs, enrichment analyses were used for functional annotation of DEARGs. Gene Ontology (GO) analysis was performed to screen the potential function of DEARGs and Kyoto Encyclopedia of Genes and Genomes (KEGG) was used for pathway analysis of DEARGs. Terms with a *p*-value< 0.05, a minimum count of 3, and an enrichment factor > 1 were collected and grouped into clusters based on their membership similarities. The enrichment analyses were performed in Metascape (http://metascape.org).

### Diagnostic value of ARGs for sarcoma

Early and accurate diagnosis for sarcoma is vital for systematic treatment. Although the diagnostic role of ARGs for several diseases were reported, the value for sarcoma remains unclear [20, 21]. In the present study, several DEARGs were incorporated into the analysis of diagnostic value of ARGs. First, to avoid overfitting among DEARGs, Least absolute shrinkage and selection operator (LASSO) analysis was performed and significant genes were incorporated into receiver operating characteristic (ROC) curve analysis. The area under the curve (AUC) value was used to quantify the diagnostic value of AGRs. Furthermore, to validate the diagnostic value of AGRs for sarcoma patients. The ROC curves of diagnostic genes in two validate cohorts were generated and corresponding AUC values were determined.

### Characteristic of autophagy-related phenotypes

Based on the DEARGs, ConsensusClusterPlus package was used to perform the unsupervised clustering analysis to explore the distinct autophagy patterns of sarcoma. In addition, “CIBERSORT” package was used to quantify the 22 types immune cells and the “ESTIMATE” package was used to determine the immune and stromal scores. The difference of clinical data, DEARSs, tumor microenvironment score, microsatellite instability, and immune cell fraction between clusters were analyzed.

### Construction of ARG signatures for OS and DFS of sarcoma patients

Previous studies indicated that AGRs-based signature can serve as effective biomarkers for predicting patient’s prognosis [22–25], but the role in sarcoma patients remains unclear. In the present study, two ARGs-based signatures were generated to predict the OS and DFS for sarcoma patients, respectively. Firstly, the TCGA-SARC cohort was used as the training cohort to develop signatures. Based on DEARGs, the univariate Cox regression model was performed. Genes with a *P* value< 0.05 was considered to be statistically significant. Then, LASSO regression was performed to exclude overfitting genes. The significant genes in the LASSO analysis were further incorporated into the multivariate Cox regression model to develop signatures, and the risk scores of each patient were calculated by combining regression coefficient and gene expression. According to the median of risk score, 259 sarcoma patients were divided into low- and high-risk groups. The performance of two signatures was evaluated by Kaplan-Meier survival curve and the time-dependent ROC curves.

### External validation of ARG signatures

External validation by another independent cohort is vital for prognostic signature. Therefore, we downloaded two independent cohorts (TARGET-OS and GSE30929) to validate our signatures. The risk score of patients in TARGET-OS were calculated by the OS signature, whereas the risk score of patients in GSE30929 were calculated by the DFS signature. The time-dependent ROC curves were generated for two validation cohorts. In addition, survival curves were generated to show the prognostic difference between low- and high-risk groups.

### Development of ARG-clinical nomogram for sarcoma

Clinical variables, such tumor site, age and metastatic disease, were also important prognostic biomarkers for sarcoma patients. Therefore, to enhance the predictive ability of signature, we intended to establish two ARG-clinical nomograms. First, the univariate Cox analysis was performed to select OS- and DFS-related variables. Significant variables in the univariate Cox analysis were further incorporated into the multivariate Cox analysis to determine independent prognostic clinical biomarkers. Then, two nomograms that included corresponding independent prognostic biomarkers were developed by “rms” package. The concordance index(C-index) was calculated to show the discrimination and the calibration curve was used to show the calibration of nomogram.

## Results

### Profiling of DEARGs in sarcoma patients

**Fig. 1:**
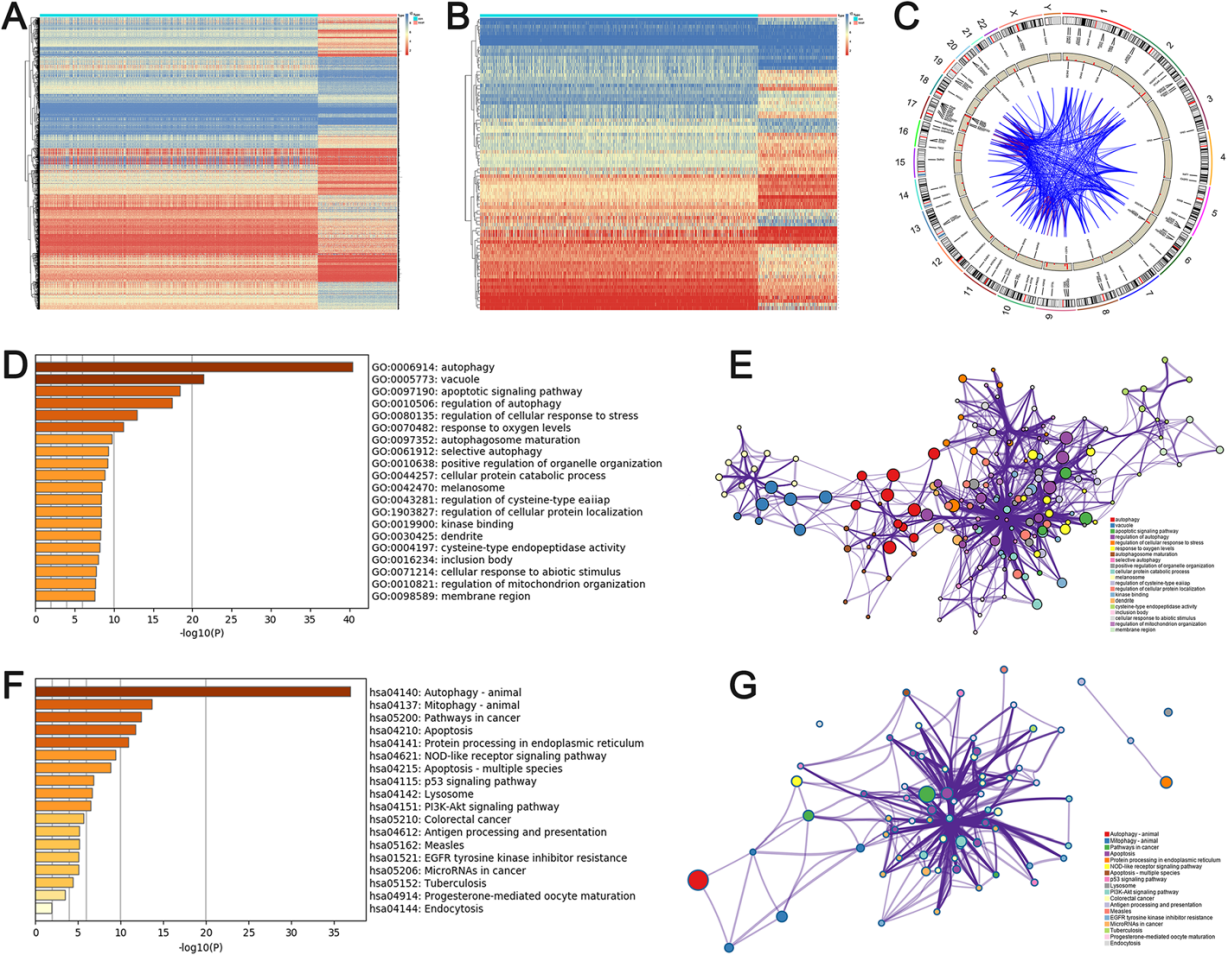
Analysis of differential genes in patients with sarcoma. **a** Genes that are misexpressed in sarcomas. **b** Screened differential genes in sarcomas. **c** The position of differential genes on chromosomes. **d** and **e** GO enrichment analysis of differential genes. **f** and **g** KEGG enrichment analysis of differential genes

The differential analysis was performed between 911 normal samples and 259 sarcoma samples. Totally, 5609 genes were dysregulated in sarcoma samples (Fig. 1a). By matching the list of ARG from HADb, 84 DEARGs were determined (Fig. 1b). Among 84 DEARGs, 40 genes were highly expressed in sarcoma samples. The location of 84 DEARGs on chromosomes are showed in Fig. 1c.

To further understand the molecular mechanism of DEARGs, we performed GO and KEGG annotation analyses. The GO analysis showed that DEARGs were mainly involved in “autophagy”, “vacuole”, “apoptotic signaling pathway”, “regulation of autophagy”, and “regulation of cellular response to stress” (Fig. 1d and e). Furthermore, KEGG analysis demonstrated that the pathway of DEARGs were mainly involved in “Autophagy – animal”, “Mitophagy – animal”, “Pathways in cancer”, “Apoptosis”, and “Protein processing in endoplasmic reticulum” (Fig. 1f and g). Generally, both GO and KEGG analyses showed these 84 DEARGs were significantly associated with autophagy and malignance.

### DEARGs are robust diagnostic biomarkers for sarcoma

**Fig. 2:**
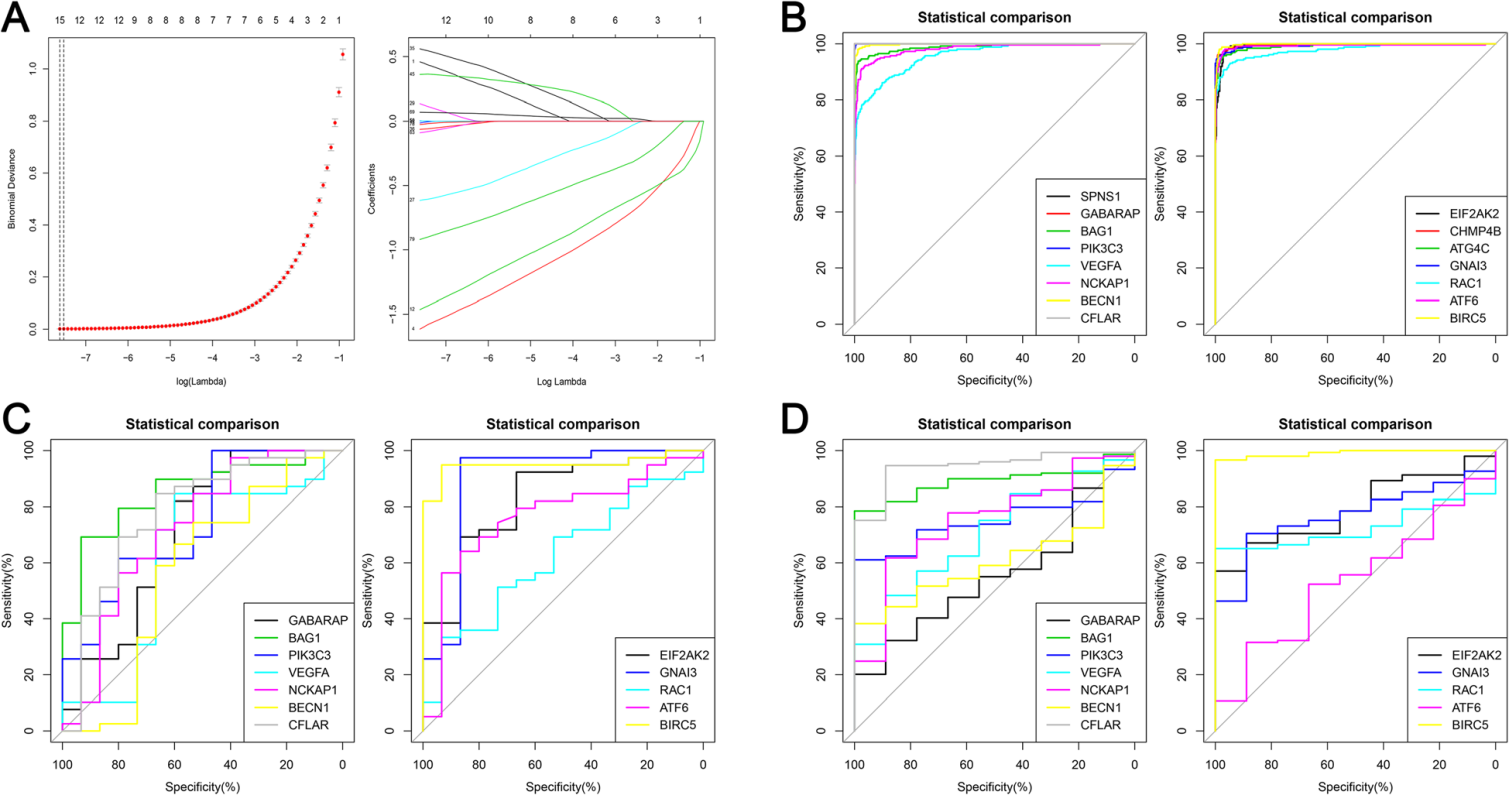
Diagnostic value of ARGs for sarcoma. **a** The results of LASSO analysis; **b** The diagnostic ROC curves of independent ARGs in TCGA cohort; **c** The diagnostic ROC curves of independent ARGs in the GSE2719 cohort; **d** The diagnostic ROC curves of independent ARGs in the GSE21122 cohort

Based on 84 DEARGs and LASSO analysis, 15 DEARGs were confirmed (Fig. 2a). The ROC curves and corresponding of these genes are shown in Fig. 2b. The AUC values of aforementioned genes were higher than 0.950, which means these genes have terrific discrimination between sarcoma and normal samples. Furthermore, the expression 12/15 genes were obtained from GSE2719 and GSE21122 cohorts. For the GSE2719 cohort, 9/12 genes were confirmed as diagnostic biomarkers for sarcoma (AUC > 0.500 and *P* values< 0.05), with AUC values range from 0.709–0.950 (Fig. 2c). Additionally, in the GSE21122 cohort, 9/12 genes were determined as diagnostic genes for sarcoma (AUC > 0.500 and *P* values< 0.05), with AUC values range from 0.704–0.991 (Fig. 2d). Interestingly, seven genes were successfully validated in both GSE2719 and GSE21122 cohorts and BIRC5 has highest AUC values in in both cohorts. Therefore, we speculated that BIRC5 may be a robust diagnostic and therapeutic biomarker for sarcoma.

### ARG-based clusters associated with tumor microenvironment

**Fig. 3:**
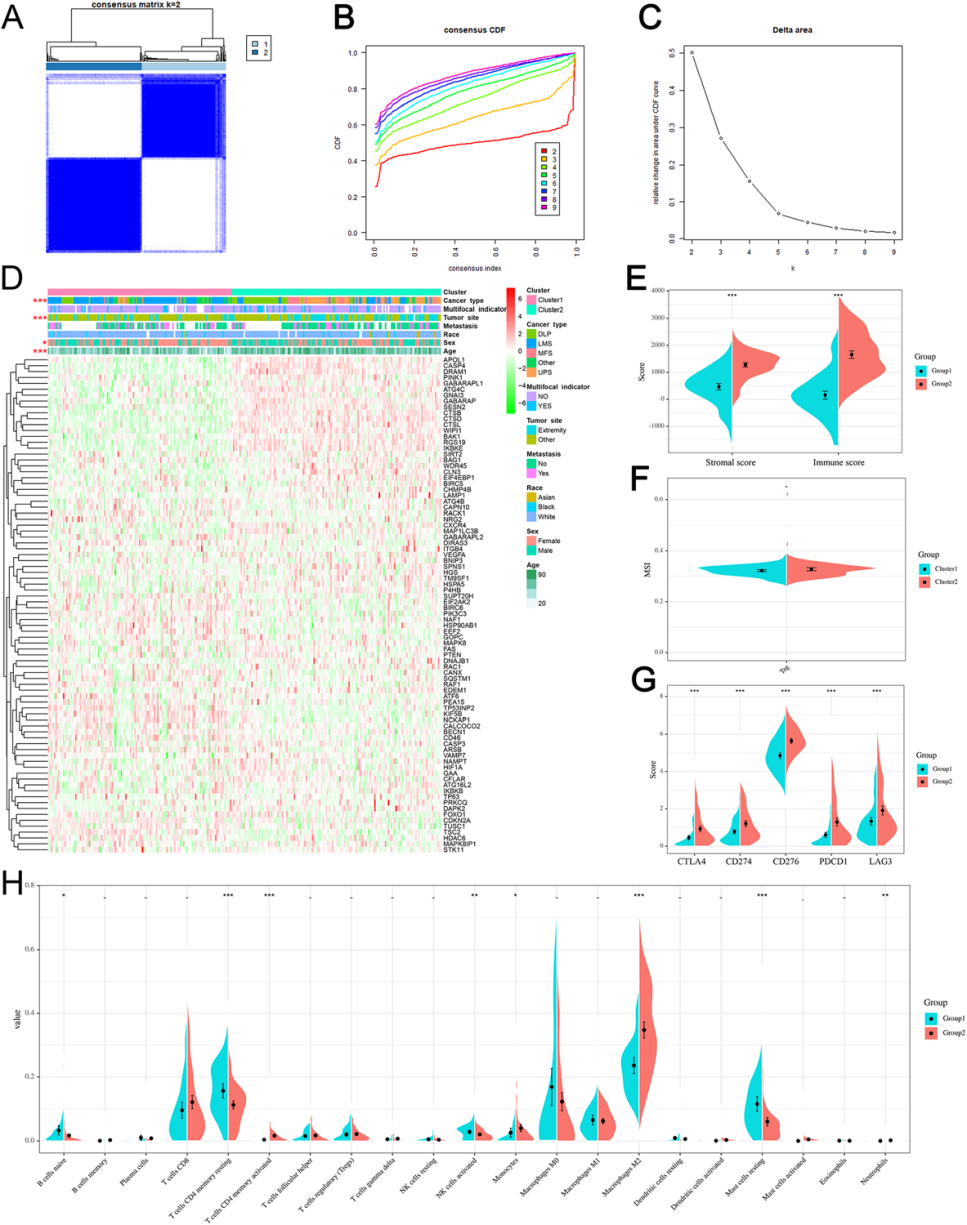
Two different ARG clusters are significantly related to TME characteristics. **a**-**c** Consensus matrix heatmap defines two different ARG clusters of sarcoma patients. **d** The heat map shows the differences between different clusters and clinical features (tumor site, histological type, sex, and age). **e** The violin plot shows the immune and stromal scores between the two clusters. **f** MSI in sarcoma of two ARG clusters. **g** Difference in expression of immune checkpoints between two clusters. **h** The difference of 21 kinds of immune cells between the two groups in patients with sarcoma

By performing unsupervised cluster analysis, 259 sarcomas were divided into two clusters (Fig. 3a-c). Tumor site, histological type, sex, and age were significant difference between two clusters (Fig. 3d). Specifically, leiomyosarcoma accounted for the largest proportion in the cluster1, accounting for 62.8%. In the cluster2, undifferentiated pleomorphic sarcoma and dedifferentiated liposarcoma were the main components, accounting for 31.9 and 28.3% respectively (Fig. 3d). Moreover, tumor microenvironment analysis further showed the heterogeneity of two clusters. Compared with cluster 2, cluster 1 had lower immune and stromal scores (Fig. 3e). Specifically, the fraction of B cells naive, T cells CD4 memory resting, NK cells activated, and Mast cells resting were significantly higher in cluster 1, whereas the fraction of T cells CD4 memory activated, Monocytes, Macrophages M2, and Neutrophils were significantly lower in cluster 1(Fig. 3h). In addition to immune cells, we also find that the expression of common immune check points was significantly different between two clusters (Fig. 2g) but there were no significant difference of MSI (Fig. 3f).

### Construction two favorable ARG signatures of sarcoma patients

**Fig. 4:**
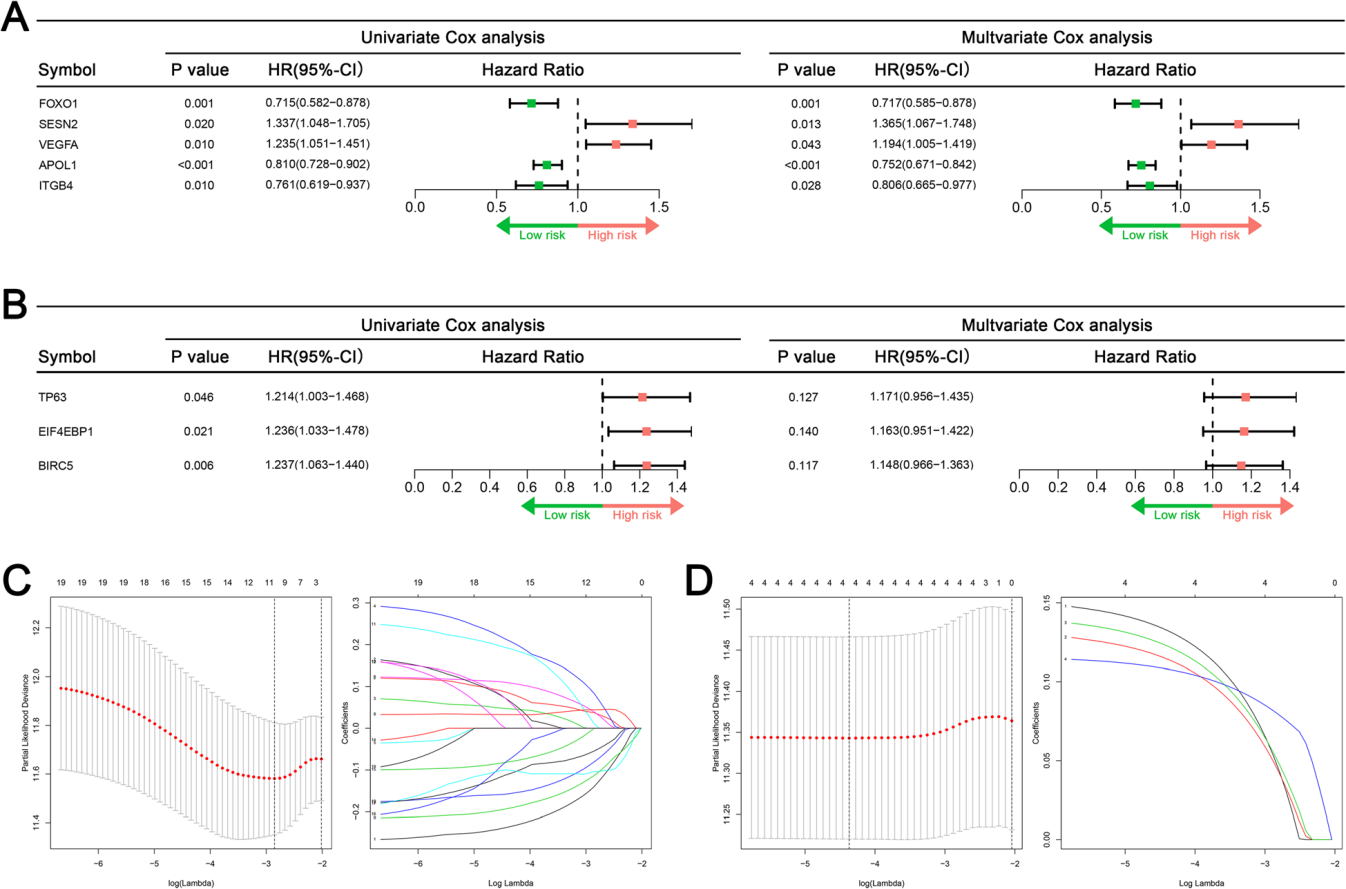
Screening ARG related to prognosis. **a** Univariate and multivariate Cox regression were used to screen OS-related ARG. **b** Univariate and multivariate Cox regression were used to screen DFS-related ARG. **c** LASSO were used to screen OS-related ARG. **d** LASSO were used to screen DFS-related ARG

**Fig. 5:**
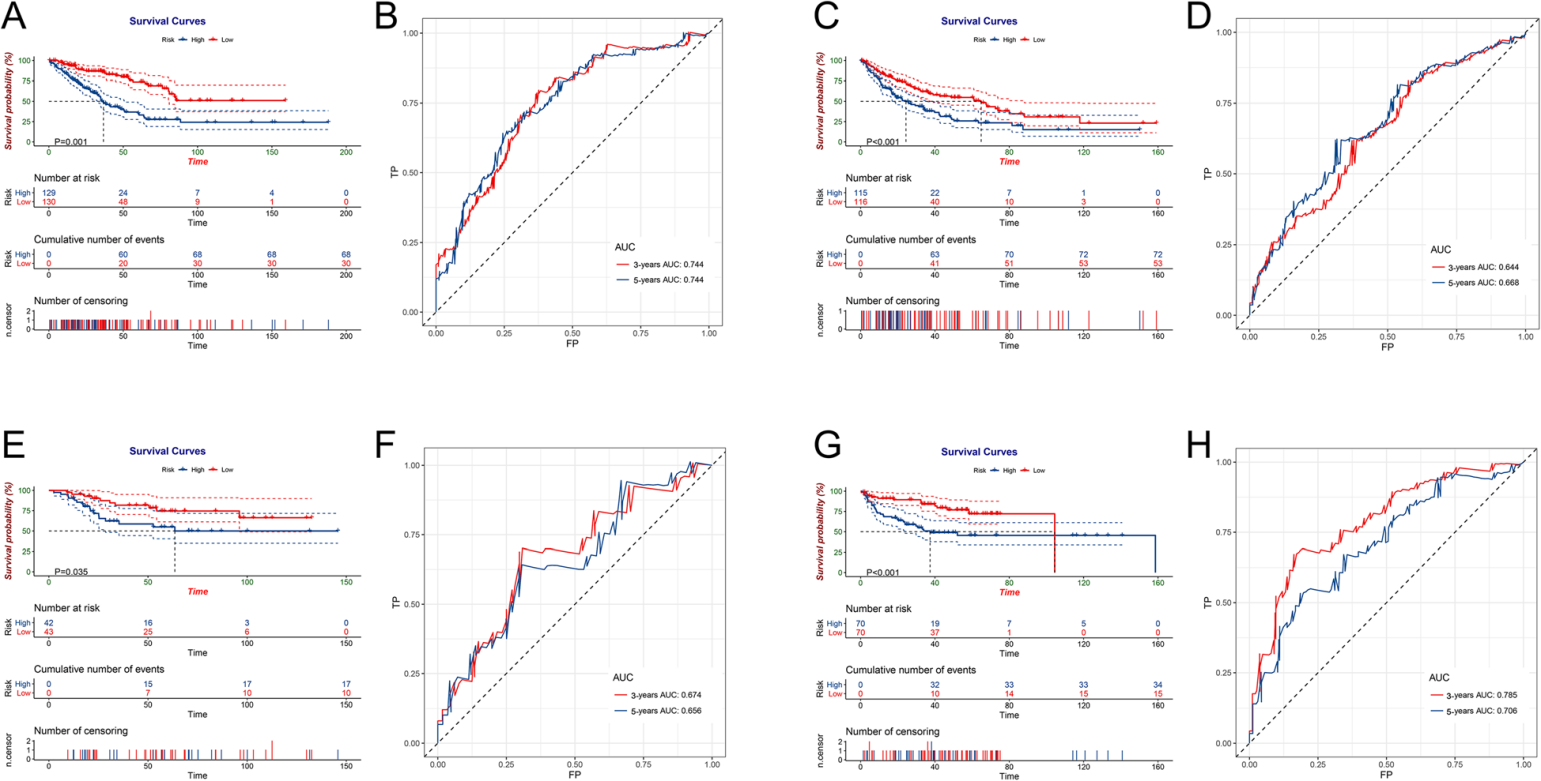
Survival analysis of patients with sarcoma. **a** Survival curve related to OS of sarcoma patients. **b** ROC curve related to 3- and 5-year OS in patients with sarcoma. **c** and **d** Survival curve and ROC curves related to 3- and 5-year DFS in patients with sarcoma. **e** and **f** TARGET-OS queues are used to verify OS signatures. **g** and **h** GSE30929 queues are used to verify DFS signatures

Univariate Cox regression found that 17 DEARGs were significantly related to the OS and 4 DEARGs were related to the DFS. Subsequently, in the LASSO analysis, seven OS-related DEARGs were exclude but no DFS-related genes were excluded (Fig. 4c and d). Finally, by performing multivariate Cox analyses, five and three DEARGs were incorporated into OS and DFS signatures, respectively (Fig. 4a and b). According to the median of risk score, all patients were divided into low- group and high-risk groups. Kaplan-Meier survival curve analyses showed that the prognosis of OS and DFS in high-risk group was significant worse than low-risk patients (Fig. 5a and c). The ROC curves also indicated favorable discrimination of signature. The 3- and 5-years AUC values of OS signature were both 0.744 (Fig. 5b), and the AUC values of DFS signature at 3- and 5-years were 0.644 and 0.668, respectively (Fig. 5d).

### ARG signatures showed stable prognostic value in independent cohorts

To verify the accuracy of two prognostic signatures, we calculated the risk score of each patient in the corresponding validation cohort. The TARGET-OS cohort was used to validate OS signature and the survival analysis showed that low-risk patients were favorable OS (Fig. 5e). The ROC curves at 3- and 5-years also have favorable discrimination, with AUC were 0.674 and 0.656, respectively (Fig. 5f). Meanwhile, the GSE30929 cohort was used to validate the DFS signature and the verification result also confirmed that the DFS signature based on ARGs was a stable prognostic prediction tool (Fig. 5g and h).

### Development of nomograms for predicting 3- and 5-years prognosis for sarcoma patients

**Fig. 6:**
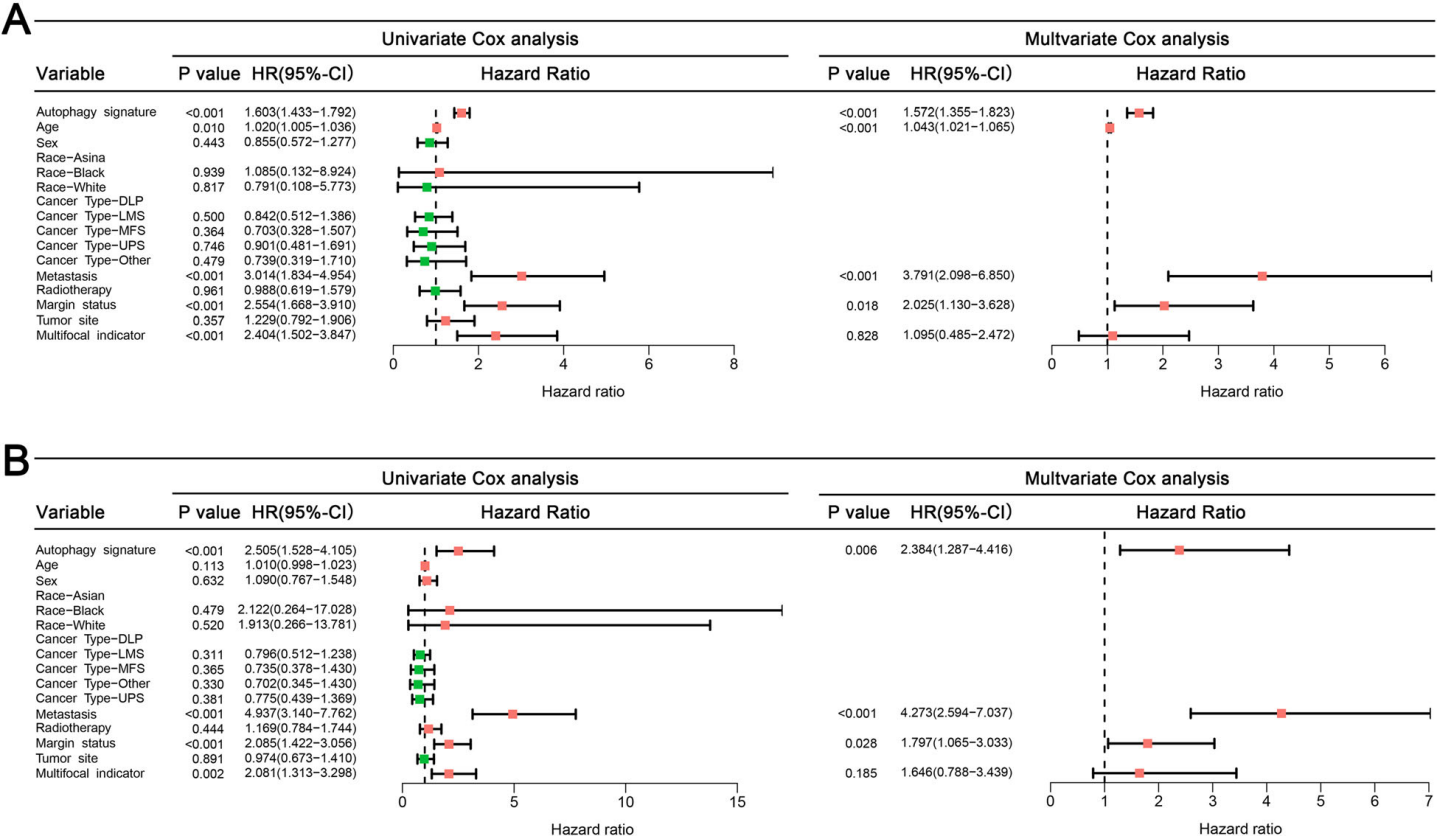
Screening of clinical independent variables based on ARG-related characteristics. **a** Univariate and multivariate COX regression was used to screen independent OS-related variables. **b** Univariate and multivariate COX regression was used to screen independent DFS-related variables

**Fig. 7:**
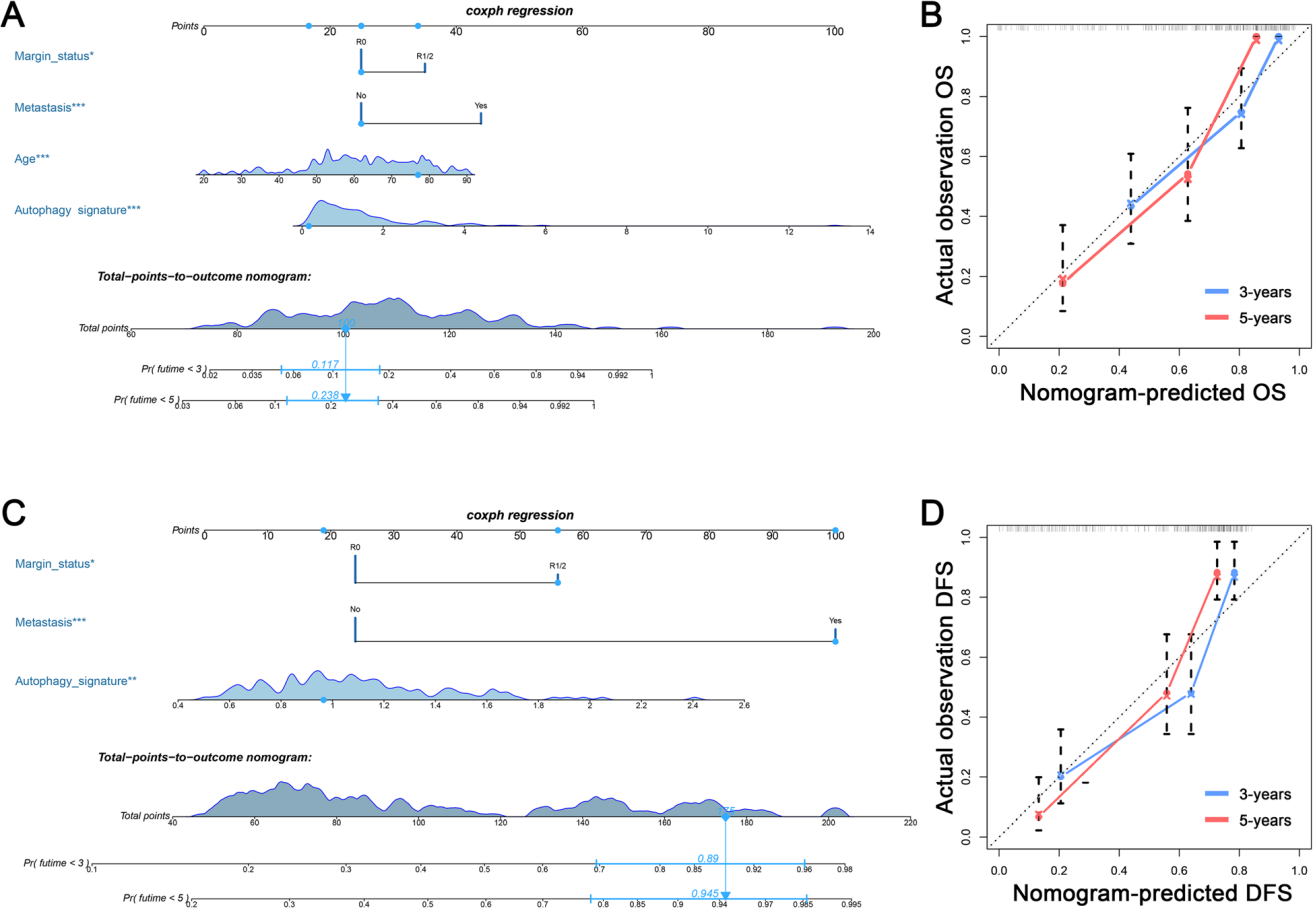
Construction and verification of novel nomogram based on the ARG-related signature and independent clinical variables. **a** A novel nomogram for predicting 3-and 5-year OS of sarcoma patients. **b** Calibration curves of the nomogram at 3-, 5-years. **c** A novel nomogram for predicting 3-and 5-year DFS of sarcoma patients. **d** Calibration curves of OS-related nomogram in 3-, 5-years

The Cox analysis for clinical data and ARG signatures are illustrated in Fig. 6. Generally, both OS and DFS signatures were independent prognostic biomarkers for sarcoma (Fig. 6a and b). For clinical data, age, metastatic status, and margin status were confirmed as independent OS-related variables (Fig. 6a), whereas metastasis and margin status were DFS-related variables (Fig. 6b). Two nomograms were developed (Fig. 7a and c). The values of C-index of OS and DFS nomograms were 0.818 and 0.747, respectively. The calibration plots of the both nomograms showed that the nomogram-predicted outcomes were in good agreement with the observational outcomes (Fig. 7b and d). Generally, these results showed that ARG-clinical nomograms can accurately predict the OS and DFS of sarcoma patients.

## Discussion

Sarcoma is a primary malignant tumor derived from mesenchymal tissue [26]. Studies have confirmed that autophagy had impact on the tumorigenesis and progression of sarcoma [27]. In this study, we comprehensively analyzed the role of ARGs in sarcoma patients. A total of 84 genes were determined as DEARGs in sarcoma, which were involved in several autophagy- and tumor-related functions and pathways. Further diagnostic analysis indicated that several ARGs were robust biomarkers for sarcoma diagnosis and survival analysis confirmed the prognostic value of DEARG. One OS autophagy signature and another DFS autophagy signature were developed and validated in independent cohorts. In addition to survival analyses, we firstly performed the ARG-based cluster analysis for sarcoma. Two clusters were determined and showed significantly associated with clinical variables and tumor microenvironment.

Compared with previous studies, this study has the following advantages. First of all, based on the identified DEARG, we not only analyzed the survival of patients with sarcoma, constructed an autophagy-related risk model, but also analyzed the immune microenvironment of sarcoma. Through multi-angle and multi-level analysis, we have a deeper understanding of the prognosis of sarcoma. Secondly, we identified 5 biological functions and pathways related to autophagy in sarcoma, which have been proved to be significantly related to the occurrence and development of malignant tumors. Then, for the first time, our team identified and verified the autophagy-related characteristics of sarcoma from three different databases: GEO, TCGA and TARGET. Finally, we developed two nomograms to predict 3- and 5-year OS and DFS in sarcoma patients and found that it had good predictive performance. Therefore, through the research and analysis of the autophagy-related prognostic model of OS and DFS in, we can provide individual survival prediction and promote the choice of better treatment strategies, which is of guiding significance in clinical application.

Consistent with previous studies, all the genes we identified in ARGs that predict the prognosis of patients with sarcomas have been reported to be closely related to the prognosis of sarcomas or other malignant tumors in previous studies. As early as 1999, some scholars proposed that vascular endothelial growth factor (VEGF) is related to the poor prognosis of osteosarcoma [28]. VEGFA, a member of the VEGF family, is one of the key regulators of tumor angiogenesis and plays a role in cancer by stimulating VEGF receptor (VEGFR) on tumor cells [29]. At present, drugs targeting VEGF receptors have been widely used in the treatment of sarcoma, colon cancer, breast cancer and other cancers [30, 31]. Mika et al. Found that the expression of BIRC5/survivin decreased and sarcoma cells died after YM155 anti-cancer treatment experiment on human synovial sarcoma cell line and gene engineering mouse model of synovial sarcoma [32]. Moreover, Wirries A et al. [11] reported that survivin and its downstream target Bcl-2 were suppressed by Panobinostat in osteosarcoma cells, which provide new perspectives for the therapy of osteosarcoma. The above research results also support our analysis hypothesis that the expression of VEGFA and BIRC5 is too high, the prognosis of patients may be poor. Niedan et al. found that high expression of FOXO1 induces cell death in Ewing’s sarcoma through WS-FLI1 inhibitory signals [33]. In addition to sarcomas, high expression of APOL1 can induce autophagy and autophagy-related cell death, which may be the key to maintaining cell homeostasis in the kidney. Similarly, APOL1 protects renal cells from renal cell carcinoma [34]. Rutkovsky et al. have shown that the overexpression of EIF4EBP1 in patients with breast cancer is associated with poor prognosis. Silence of EIF4EBP1 can significantly inhibit the proliferation of breast cancer cells by promoting G1 cell cycle arrest [35].

In addition, through functional enrichment analysis, we identified five biological functions of “autophagy”, “vacuole”, “apoptosis signal pathway”, “autophagy regulation” and “cellular stress response regulation”, as well as “autophagy-animal”, “mitosis-animal”, “cancer pathway”, “apoptosis” and “protein processing in endoplasmic reticulum”. The five autophagy signal pathways were significantly enriched in the sample DEARG. We speculate that the imbalance of expression of these functions and signaling pathways related to autophagy may be related to the biological pathway of sarcoma.

However, there are also some limitations in this study. Firstly, the data used in the study are taken from several public databases, from which the clinical information downloaded is limited and incomplete. Therefore, some important prognostic factors, such tumor grade, tumor size, surgery information, and distant metastatic data, were not available and were not included in the present study. Secondly, the muscle and fat tissues were selected to form as normal tissue to compare the sarcoma. However, some sarcomas, such as myxofibrosarcoma and synovial sarcoma, are not derived from these tissues in the first place. Therefore, this may also lead to some bias in the present study. Finally, as a regulatory factor in the occurrence and development of sarcoma, the molecular mechanism of ARGs regulation needs to be confirmed by further research, so as to promote the clinical application of ARGs as a prognostic marker and therapeutic target of sarcoma.

## Conclusions

To sum up, in this study, we obtained 8 autophagy-related genes related to the prognosis of patients with sarcoma. By evaluating the expression profile of these genes, we constructed an autophagy-related risk score model and proved that it can be used as an independent prognostic factor for patients with sarcoma. The model is of great value in predicting the prognosis of patients with sarcoma. and can indicate the therapeutic target of sarcoma. Then, we established a novel prognostic nomogram, model, which combines autophagy-related characteristics and clinical parameters, which can provide individual survival prediction and promote the choice of better treatment strategies.

## Data Availability

The datasets generated during and/or analysed during the current study are available from the corresponding author on reasonable request.

## Abbreviations

ARGs: Autophagy-related genes
TCGA: The Cancer Genome Atlas
GTEx: Genotype-Tissue Expression
DEGs: Differentially Expressed Genes
DEARGs: Differentially expressed ARGs
TME: Tumor Microenvironment
OS: Overall survival
DFS: Disease-free survival
GO: Gene Ontology
KEGG: Kyoto Encyclopedia of Genes and Genomes
ROC: Receiver Operating Characteristic
AUG: Area Under the Curve
LASSO: Least absolute shrinkage and selection operator
